# UBE2C Drives Human Cervical Cancer Progression and Is Positively Modulated by mTOR

**DOI:** 10.3390/biom11010037

**Published:** 2020-12-30

**Authors:** An-Jen Chiang, Chia-Jung Li, Kuan-Hao Tsui, Chung Chang, Yuan-chin Ivan Chang, Li-Wen Chen, Tsung-Hsien Chang, Jim Jinn-Chyuan Sheu

**Affiliations:** 1 Institute of Biomedical Sciences, National Sun Yat-sen University, Kaohsiung 804, Taiwan; ajchiang490111@gmail.com; 2 Department of Obstetrics and Gynecology, Kaohsiung Veterans General Hospital, Kaohsiung 813, Taiwan; nigel6761@gmail.com (C.-J.L.); khtsui60@gmail.com (K.-H.T.); zsedc515157@gmail.com (L.-W.C.); 3 Institute of BioPharmaceutical Sciences, National Sun Yat-sen University, Kaohsiung 804, Taiwan; 4 Department of Applied Mathematics, National Sun Yat-sen University, Kaohsiung 804, Taiwan; cchang@math.nsysu.edu.tw; 5 Institute of Statistical Science, Academia Sinica, Taipei 115, Taiwan; ycchang@sinica.edu.tw; 6 Department and Graduate Institute of Microbiology and Immunology, National Defense Medical Center, Taipei 114, Taiwan; 7 Department of Health and Nutrition Biotechnology, Asia University, Taichung 413, Taiwan; 8 School of Chinese Medicine, China Medical University, Taichung 406, Taiwan; 9 Department of Biotechnology, Kaohsiung Medical University, Kaohsiung 807, Taiwan

**Keywords:** UBE2C, human papillomavirus, cervical cancer, bioinformation

## Abstract

Cervical cancer is a common gynecological malignancy, accounting for 10% of all gynecological cancers. Recently, targeted therapy for cervical cancer has shown unprecedented advantages. Several studies have shown that ubiquitin conjugating enzyme E2 (UBE2C) is highly expressed in a series of tumors, and participates in the progression of these tumors. However, the possible impact of UBE2C on the progression of cervical squamous cell carcinoma (CESC) remains unclear. Here, we carried out tissue microarray analysis of paraffin-embedded tissues from 294 cervical cancer patients with FIGO/TNM cancer staging records. The results indicated that UBE2C was highly expressed in human CESC tissues and its expression was related to the clinical characteristics of CESC patients. Overexpression and knockdown of UBE2C enhanced and reduced cervical cancer cell proliferation, respectively, in vitro. Furthermore, in vivo experiments showed that UBE2C regulated the expression and activity of the mTOR/PI3K/AKT pathway. In summary, we confirmed that UBE2C is involved in the process of CESC and that UBE2C may represent a molecular target for CESC treatment.

## 1. Introduction

Cervical cancer remains an important genital tract malignancy in women, ranking fourth in both incidence and mortality for female malignancies worldwide [1,2]. In Taiwan, cervical cancer ranked as the eighth most common cancer in females in 2016, with an age-standardized incidence rate of 8.7 per 100,000 women. Nearly one-quarter of all cases of cervical cancer were diagnosed in Taiwanese women aged 30–39 [3].

Human papillomavirus (HPV) infection is considered to be the leading cause of cervical, vulvar, vaginal, anal, oropharyngeal, and penile cancer [4,5]. More than 100 types of HPV have been identified, and at least 20 are associated with cervical cancer [6,7,8]. HPV types 6 and 11 are low-risk or non-oncogenic viruses that can cause benign or low-grade cervical cell abnormalities. The oncogenic impact of HPV type 16/18 is critical; however, a recent study in Taiwan found that patients with high-grade squamous intraepithelial lesions were highly infected with high-risk HPV types other than HPV 16/18, such as HPV58, HPV52, HPV33, and HPV18 [9,10]. HPV infection triggers a variety of intracellular effects within different signaling pathways. The mammalian targets of the phosphoinositide 3-kinase (PI3K)/protein kinase B (AKT)/rapamycin (mTOR) pathway are dysregulated in gynecological cancers, especially in tumors related to HPV [11]. Factors that influence the activation and/or dysregulation of the PI3K/AKT/mTOR pathway may be potential targets for drug therapy [12,13]. The dysregulation of mTOR and other proteins related to this pathway in solid tumors can cause tumor cells to be more sensitive to mTOR inhibitors than normal cells [14]. The mechanism of pathway activation includes loss of function of the tumor suppressor gene phosphatase and tensin homolog (PTEN), PI3K amplification or mutation, AKT amplification or mutation, growth factor receptor activation, and exposure to carcinogens [15,16].

Ubiquitination is a critical cellular mechanism for targeting abnormal or short-lived proteins for degradation. The modification of proteins with ubiquitin involves at least three classes of enzymes: ubiquitin-activating enzymes, ubiquitin-conjugating enzymes (E2s), and ubiquitin-protein ligases. Ubiquitin-conjugating enzyme 2C (UBE2C) encodes a member of the E2 ubiquitin-conjugating enzyme family; the gene features multiple transcript variants encoding different isoforms. UBE2C is a member of the anaphase promoting complex/cyclosome, promoting the degradation of several target proteins along cell cycle progression, during metaphase/anaphase transition [17,18]. Overexpression of UBE2C causes chromosome mis-segregation, a loss of genomic stability, and alterations of the cell cycle profile, which are the typical characteristics of cancer [19,20]. Importantly, UBE2C transgenic mice show a broad spectrum of spontaneous tumors that demonstrate UBE2C is a prominent protooncogene [20]. UBE2C has been found overexpressed in a wide range of different solid tumors [21,22]. At present, the expression of UBE2C in cervical cancer and its relationship with HPV are still unclear. The Cancer Genome Atlas (TCGA) indicates that patients with cervical cancer with high UBE2C/cancer marker co-expression levels have a poor prognosis. In this study, we explored the potential role and clinical significance of UBE2C in cervical squamous cell carcinoma (CESC). 

## 2. Materials and Methods 

### 2.1. Ethic Statement

The retrospective study was approved by the Institutional Review Board of Kaohsiung Veterans General Hospital (VGHKS15-CT6-09) and conformed to the current ethical principles of the Declaration of Helsinki.

### 2.2. Archival Paraffin-Embedded Samples 

The paraffin-embedded tissues from 320 cervical cancer and 58 benign patients with patients were obtained from the Department of pathology at Kaohsiung Veterans general hospital. Those tissues blocks were made for pathological diagnosis during 1990 to 2014. The clinical stage and extent of the tumors were identified based on the FIGO/TNM classification system, and followed the clinical data to 2018.

### 2.3. Human Tissue Microarray (TMA) Immunohistochemical Analysis

Tumor tissues from formalin-fixed, paraffin embedded tissue blocks of carcinomas with a core size of 1.5 mm were assessed. Sampling sites including 2 tumor sites and 1 non-tumor site were marked on each donor blocks by pathology physician, and the tissue cylinders precisely arrayed into recipient blocks each with a core size of 1.5 mm. The blocks of embedded tissue for TMA were performed using a manual tissue microarrayer (Beecher Instruments, Silver Spring, MD, USA); and the recipient was incubated overnight at 37 °C before sectioning. Immunohistochemical analysis in the TMA sections was carried out as described in a previous study [22]. The TMA sections (4 μm) were deparaffinized and incubated with 10 μg/mL proteinase K (Sigma-Aldrich, St. Louis, MO, USA) at 37 °C for 30 min. Quality assurance tests and the confirmation of diagnoses were carried out by staining the TMA sections with hematoxylin and eosin (HE). The slides were treated with anti-UBE2C antibody (1:100, H00011065-M01, Abnova), anti-HPV16 E6, anti-HPV18 E6 and anti-HPV58 E6.

### 2.4. Evaluation of the Human TMA Sections

The TMA sections were examined and scored, the distribution of positive staining tumor tissue was scored as the percentage of labeled cells (0–100%); and intensity scores (negative = 0; weak = 1, moderate = 2, or strong = 3). Multiplying the percentage of labeled cells and staining intensity scores were recorded for histological scoring (H score, ranging 0–300). For the purpose of statistical analysis, tumors with H-score 0–100, 101–200, 201–300 were categorized. 

### 2.5. Cells and Reagents

Cervical cancer cell lines HeLa cells (BCRC#60005, Hsinchu, Taiwan) and CC7T/VGH cells (BCRC#60195), and CaSki cells (ATCC#CRL-1550) were used and cultured in DMEM supplemented with 10% fetal bovine serum (ThemoFisher Scientific) in a humidified atmosphere of 95% air and 5% CO_2_ at 37 °C. The UBE2C inhibitor CCI779 (Temsirolimus) was from Abcam.

### 2.6. Knockdown and Overexpression of UBE2C 

pcDNA3.1-UBE2C expression vector were generated according to previous report [22]. An amount of 2 μg pcDNA3.1-UBE2C plasmid was transfected into HeLa cells by TurboFect transfection reagent (ThermoFisher Scientific, Waltham, MA, USA). At 48 h post-transfection, overexpressed UBE2C was detected by immunoblotting. To knock down UBE2C expression in Hela cells, the cells were transfected with 2 μg UBE2C short hairpin RNA (shRNA) and control shRNA oligonucleotides for 48 h for expression analysis [22]. 

### 2.7. Cell Proliferation Assay

WST-1 assays were used to monitor the cell proliferation of HeLa cells. Cells were trypsinized and resuspended in culture medium, then plated at 5 × 10^3^ cells per well in 96-well plates and incubated for 1–3 days. The cells were then incubated with 10 μL WST-1 reagent (Roche) for 2 h. The absorbance at 450 nm was monitored and the reference wavelength was set at 620 nm.

### 2.8. Western Blot Analysis

For protein extraction, 1 × 10^6^ cells were lysed in 200 μL RIPA buffer (50 mM Tris-HCl (pH 7.5), 150 mM NaCl, 1 mM EDTA, 1% Nonidet P-40, 0.5% DOC, 0.1% SDS) containing Complete Protease Inhibitor Cocktail (Roche Applied Science, Indianapolis, IN, USA). Cell lysates were centrifuged at 14,000× *g* for 30 min at 4 °C, then supernatant was harvested. Proteins were quantified by use of the Bio-Rad DC Protein Assay kit (Bio-Rad Laboratories, Hercules, CA, USA), separated by 10% SDS-PAGE and transferred to PVDF membrane (Millipore, Billerica, MA, USA). Membranes were blotted with antibody for UBE2C (H00011065-M01, Abnova), mTOR (#2983, Cellsignal, Danvers, MA, USA.), p-mTOR (Cellsignal, #2971), AKT (Genetex, GTX121937), p-AKT (GTX128414, Genetex, Ivine, CA, USA), PI3K (Genetex, GTX112994), p-PI3K (Cellsignal, #4228), GAPDH (Genetex, GTX627408) and anti-β-actin (Thermofisher, #MA5-15739). 

### 2.9. Immunofluorescence Assay 

Cells and tissues were fixed with 4% paraformaldehyde for 30 min, then permeabilized with 0.5% Triton X-100 for 10 min. After 2 washes with phosphate buffered saline (PBS), cells were blocked with 10% skim milk in PBS. Infected cells were detected by incubation with the antibody for UBE2C (H00011065-M01, Abnova) and mTOR, then secondary anti–mouse Alexa Fluor 488 antibody (Thermo Fisher Scientific, Waltham, MA, USA). DAPI was used to stain for nuclei. Fluorescence signals were observed by fluorescence microscopy (Zeiss, Axio Observer A1, Oberkochen Germany) [23].

### 2.10. Tumor Xenograft in NOD Mice and In Vivo Bioluminescence Imaging

The animal experiments were conducted in accordance with the Animal Research: Reporting of In Vivo Experiments (ARRIVE) guidelines and approved by the institutional animal care and use committee of Kaohsiung Veterans General Hospital (IACUC: 2020-A001). Hela cells were injected subcutaneously into Female NOD-scid IL2rγ^null^ (NSG) mice at the age of 6-weeks old (5 × 10^6^ cells per injection, two injection sites per mouse). Tumor volume was measured every 3 days, and tumor size was estimated as width (mm) × length (mm) × height (mm) × 0.52. For quantification, rectangular regions of interest incorporating the entire animal were measured. The signal was measured in photons per second using Living Image software. The mice with xenograft were i.p. injection of UBEC inhibitor CCI779 (ug) every 2 days. After 21 days, the mice were sacrificed and their tumors were excised and weighed. 

### 2.11. Multi-Omics Analysis

Gene Expression Profiling Interactive Analysis (GEPIA) is a user-friendly interactive network database that can be linked and analyzed with other databases (TCGA and GTEx). Using GEPIA, we analyzed 9736 tumors and 8587 normal tissues [24]. In addition, we used UALCAN (a database that can effectively find RNAseq data in TCGA and perform gene expression and survival analysis) to analyze the expression in different clinical stages of CESC [25].

### 2.12. Statistical Analyses

To assess differences in the expression of UBE2C, HPVs clinicopathologically defined groups, Student’s *t* test for differential significance and chi-square test for categorical variables were used. A *p*-value < 0.05 was considered to indicate a statistically significant difference. GraphPad Prism (GraphPad software, La Jolla, CA, USA) and SPSS V.14.0 software (SPSS Inc., Chicago, IL, USA) were utilized to analyze the statistical differences by using *t*-test or Fisher’s exact test for two groups and one-way ANOVA for multiple group comparisons. Kaplan–Meier curves were plotted to study survival tendency, and the p value was estimated using the log-rank test. During the experiment, the investigators were blinded to the group allocation. A p value less than 0.05 was considered statistically significant. Statistical significance, * *p* value  <  0.05; ** *p* value  <  0.01; *** *p* value  <  0.001.

## 3. Results

### 3.1. Distribution and Expression of UBE2C in Cancer Tissues of Patients with CESC

**Figure 1:**
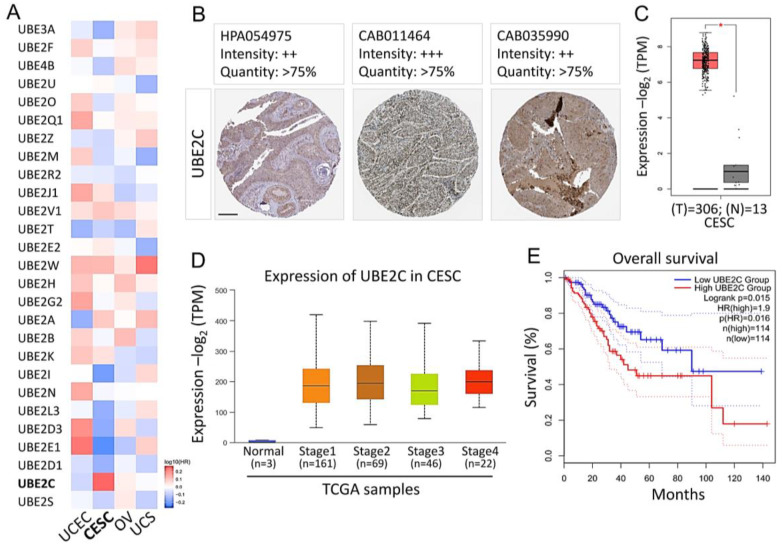
UBE2C expression in CESC and its effect on prognosis. (**A**) Heatmap representing the UBE2 family indexes of all the 27 genes across all the tissues from Web-based Gene Set analysis Toolkit. (**B**) Representative images of UBE2C IHC staining in CESC from the Human Protein Atlas (HPA). (**C**) Plots chart showing higher UBE2C expression in CESC patients. Data were achieved from TCGA. (**D**) Box plots of gene expression levels of significantly altered hub genes in CESC with different stages using UALCAN tool. (**E**) Survival rate calculated by the Kaplan–Meier survival curve in CESC patients separated according to the median expression level of each gene, using data collected from TCGA. UCEC: uterine corpus endometrial carcinoma; CESC: cervical squamous cell carcinoma and endocervical adenocarcinoma; OV: ovarian cystadenocarcinoma; UCS: uterine carcinosarcoma. * *p* < 0.05. Scale bars 200 μm.

To analyze the expression pattern of UBE2 family members in various gynecological cancers, we accessed the TCGA and GEPIA databases. We found that UBE2 is evenly distributed in a variety of human cancers, such as CESC, uterine corpus endometrial carcinoma, ovarian serous cystadenocarcinoma, and uterine carcinosarcoma. A particularly high expression of UBE2C was found in CESC (Figure 1A). In addition, we used a TMA from the Human Protein Atlas (HPA) to analyze the levels of UBE2C protein. In different databases of the HPA, we found high-grade staining for UBE2C in CESC patients (Figure 1B). Next, we determined the transcriptional expression of the target genes differentially expressed between CESC and normal tissues in TCGA. mRNA levels of UBE2C were found to be significantly increased in cervical cancer, indicating that these proteins may have potential carcinogenic effects (Figure 1C). Subsequently, to have a better understanding of the relationship between UBE2C and clinicopathological features, we analyzed the UBE2C expression level in different pathological stages by UALCAN. The results showed that the average expression level of UBE2C exhibited an upward trend with development of the TNM pathology stage (Figure 1D). In addition, CESC patients with high levels of UBE2C mRNA expression had low overall survival (Figure 1E).

### 3.2. TMA Analysis of UBE2C and HPV Expression

**Figure 2:**
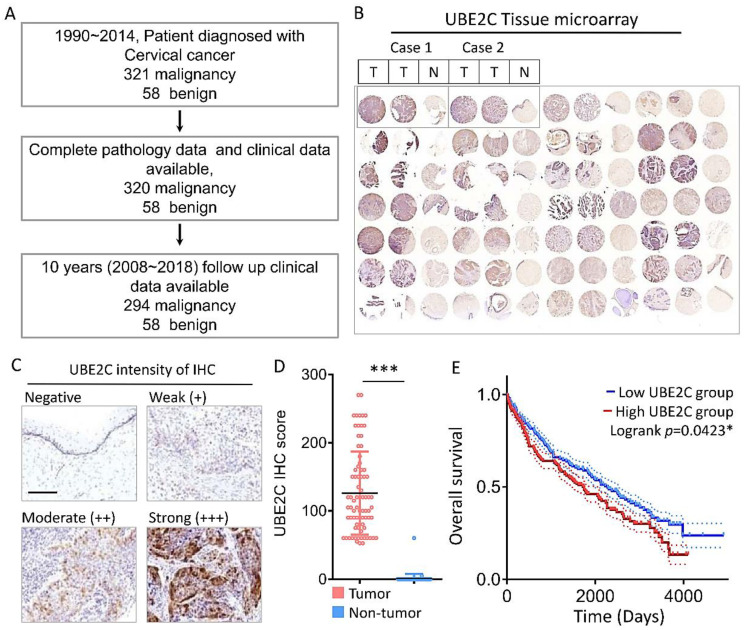
Diagnostic value of UBE2C expression in tumor biopsies during the development of CESC. (**A**) Schematic diagram of clinical recruitment for cervical cancer. (**B**) Comparative representative photomicrographs of UBE2C between normal and CESC tissues. (**C**) The representative images for negative, weak (+), moderate (++) and strong (+++) staining of UBE2C in CESC tissues. (**D**) The IHC score of UBE2C expression in CESC tissue and matched adjacent normal tissue. (**E**) Kaplan-Meier survival curves for disease-specific survival according to UBE2C expression status. * *p* < 0.05; *** *p* < 0.001. Scale bars 100 μm.

From 1990 to 2014, a total of 320 cervical cancer patients and 58 benign patients were diagnosed at a medical center in southern Taiwan. These patients were followed and their data recorded over 10 years from 2008~2018, and subsequently enrolled in this study. After excluding the cases with incomplete pathology and clinical data, 294 cervical cancer and 58 benign patient samples were subjected to TMA analysis (Figure 2A). For each patient sample block, two tumor tissue spots and one normal control spot were used for TMA; IHC analysis of UBE2C is shown as an example (Figure 2B). The IHC staining intensity of UBE2C was scored as negative = 0, weak = 1, moderate = 2, or strong = 3 (Figure 1C), and this scoring was multiplied by the percentage of positive IHC-stained cells to gain a histological score (H-score) for further analysis. The H-scores of the TMA samples showed that 89.4% (263/294) of the tumor tissue (T) samples were UBE2C positive, and expression of UBE2C was significantly higher in cervical tumor (T) tissues than in non-tumor (N) tissues (Figure 1D). Similar to the above-mentioned TCGA data, the overall survival rate of CESC patients with high UBS2C mRNA expression levels was lower than that of CESC patients with low UBS2C mRNA expression levels (Figure 2E).

**Figure 3:**
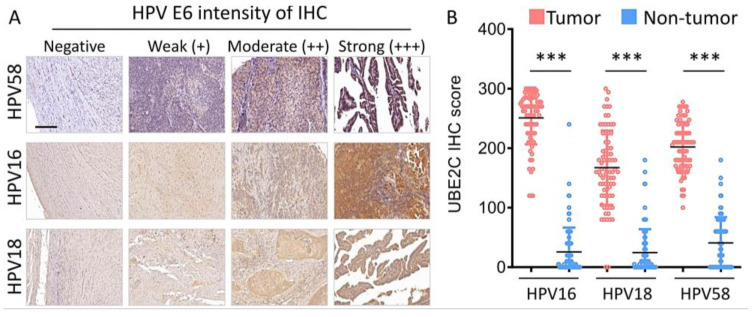
Immunoreactivity of UBE2C expression correlative with HPV expression in CESC. (**A**) The representative images for negative, weak (+), moderate (++) and strong (+++) staining of UBE2C in HPV expression. (**B**) The IHC score of UBE2C expression in HPV patients. *** *p* < 0.001. Scale bars 100 μm.

The causal role of HPV infection in cervical cancer has been convincingly confirmed. This association exists in almost all cases of cervical cancer in the world. We further analyzed whether UBE2C expression was also related to HPV. The IHC staining intensity of HPV58 E6, HPV16 E6, and HPV18 E6 was scored (Figure 3A). Compared with the benign (B) and non-tumor part (N) of the tissue, expression of HPV58, HPV16 E6, HPV18 E6, and HPV18 E6 was significantly higher in cervical tumor (T) tissue (*p* < 0.05). These data are reasonable because a pair of tumor and normal tissues were from the same patient tissue block (Figure 3B). Interestingly, HPV58 E6 expression was also detected in the non-tumor tissues of cervical cancer patients. In addition, HPV co-infection was found in our cases, which is supported by previous reports [26,27].

### 3.3. Correlation of UBE2C Expression and HPV Expression

**Figure 4:**
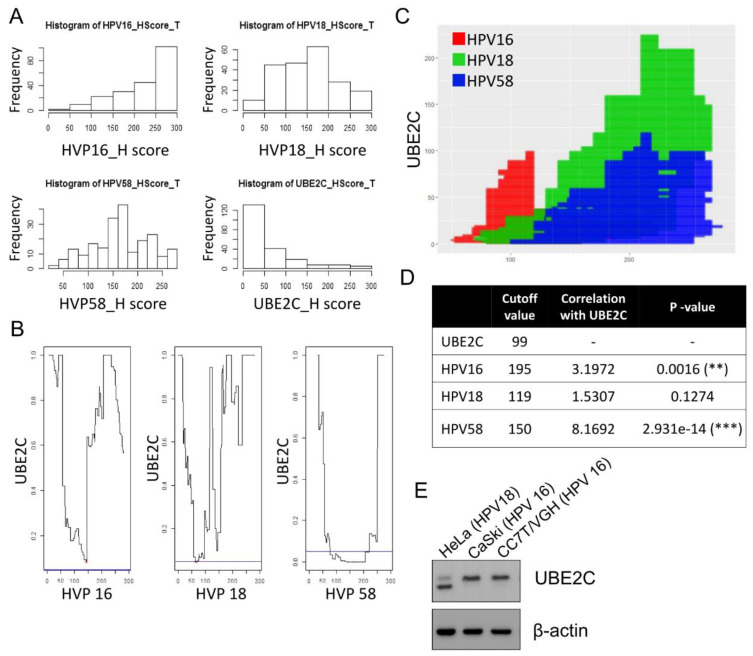
The correlation between HPV and UBE2C in CESC. (**A**) H-score distribution diagram of HPV16, 18, 58 and UBE2C. (**B**) Analysis diagram of HPV16, 18 and 58 cut points and UBE2C. (**C**) Cut-off point diagram of UBE2C and HPV, red is HPV18, green is HPV58, and blue is HPV16. (**D**) Analysis of the relationship between HPV and UBE2C. (**E**) UBE2C levels in HeLa, CaSki and CC7T cells were detected by Western blotting. ** *p* < 0.01, and *** *p* < 0.001.

To evaluate the implication of UBE2C in clinical practice, the correlation of UBE2C expression and clinical characteristics of patients with cervical cancer was analyzed with Pearson’s chi-squared and correlation tests (Figure 4 and Supplementary Table S1). Compared with tumor pathology data, the UBE2C expression level was not associated with tumor FIGO and TNM staging and tumor size, but it was significantly correlated with cervical cancer cell type (*p* < 0.05). 

The expression of tumor markers CA125, CA19-9, CEA, and SCC was not correlated with the UBE2C H-score (Supplementary Table S2). Furthermore, we analyzed the correlation between UBE2C IHC levels with different H-score groups of HPV E6 (0, 1–100, 101–200, and 201–300); the data showed that the expression of HPV58 E6, but not HPV16 or HPV18 E6, significantly correlated with UBE2C expression (*p* < 0.001) (Supplementary Table S2). Nevertheless, the correlation of UBE2C level with HPV16, 18, and 58 E6 expression was demonstrated by using a statistical approach, suggesting that HPV infection might be a crucial factor in UBE2C induction. To understand the role of UBE2C in HPV positive cervical cancer cells, we measured the expression of UBE2C in HeLa cells (HPV18 genome), and CaSki and CC7T/VGH cells (both contain the HPV16 genome) by immunoblotting (Figure 4E). We found that a short isoform protein of UBE2C exists in HeLa cells, but not in the other cell types.

### 3.4. Predicted Functions and Pathways of UBE2C in CESC

**Figure 5:**
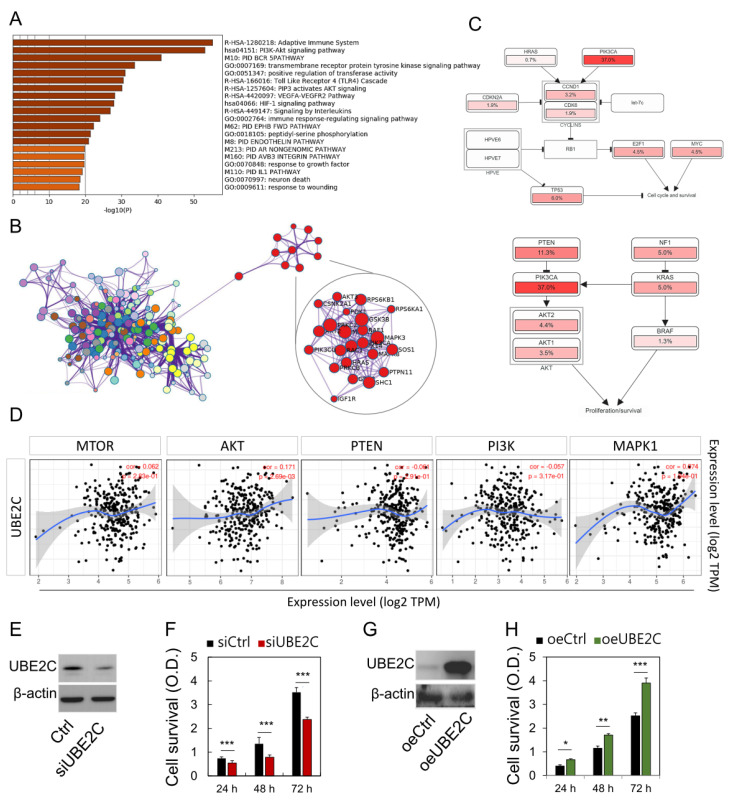
Network and biological pathway analysis of pathway analysis and protein–protein interactions. (**A**) The Metascape analysis shows the top 20 clusters of enriched sets. Left panel, heatmap of the 20 enriched terms. (**B**) Representative Molecular Complex Detection network node showing DEG regulated by UBE2C. (**C**) UBE2C and PIK3CA-TP53 pathways, identified by curated analysis, and the proliferation pathway, cell cycle progression, cell survival pathway identified by cBioPortal for Cancer Genomics. (**D**) Correlation analysis between UBE2C and mTOR, AKT, PI3K, PTEN, MAPK expression in tumors prepared with mRNA available in TCGA. (**E**–**H**) Loss-of-function and gain-of-function protein expression and cancer cell survival rate. * *p* < 0.05, ** *p* < 0.01, and *** *p* < 0.001.

We further analyzed the kappa statistics of gene members and found the remaining important terms were hierarchically aggregated into clusters. Metascape analysis revealed the first 20 clusters enriched (Figure 5A). These genes show enrichment in the molecular function categories of mTOR/PI3K/AKT signaling pathways. More specifically, each link is represented by a red circular node, the size of which is proportional to the number of input genes in the term (Figure 5B, nodes of the same color belong to the same cluster). Analysis of the frequency of known database cancer related modulation pathways with one or more mutations, copy number changes, or gene expression changes, revealed the PI3K/AKT/PTEN pathways to be regulated, respectively (Figure 5C). Furthermore, UBE2C expression was found to be correlated with mTOR, AKT, PTEN, PI3K, and MAPK expression in the TCGA-COAD dataset (Figure 5D). After analysis of multi-omic databases, it is speculated that UBE2C may affect the proliferation and survival of cancer cells. Therefore, we further investigated the regulation of UBE2C cell proliferation through *UBE2C* knockdown and overexpression methods, which reduced and enhanced cell proliferation, respectively (Figure 5E–H). Taken together, these data highlight the association between mTOR/PI3K/AKT/PTEN expression and UBE2C activation in CESC. 

### 3.5. UBE2C as a Potential Anti-Cancer Target in Vivo

**Figure 6:**
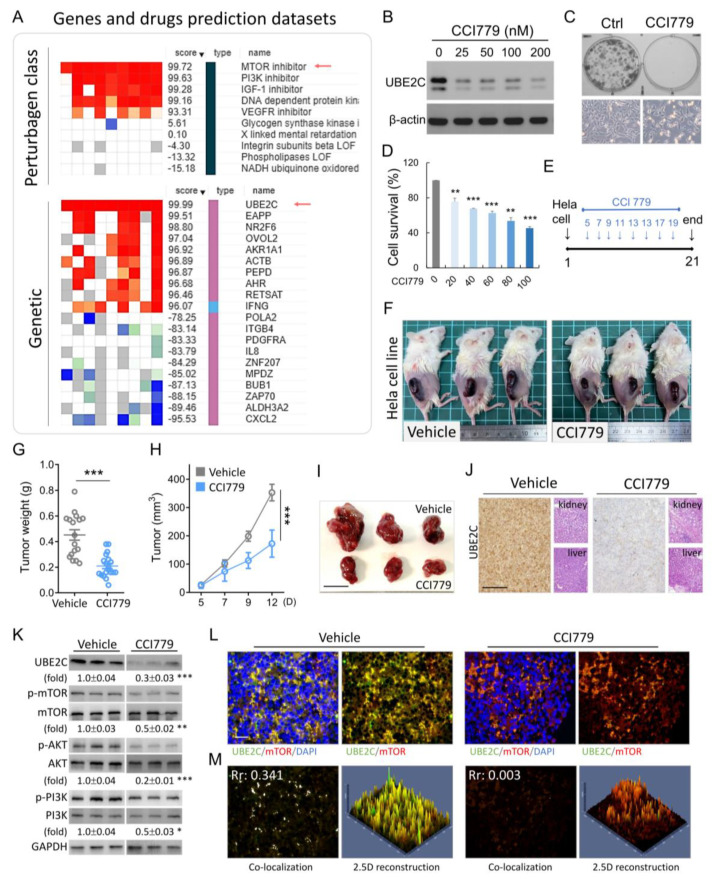
mTOR inhibitor exhibited anti-tumor activities in vitro and in vivo. (**A**) Comparison of gene expression and drug activity in CESC using CLUE (gene expression and drug activity) bioinformation database. CCI779 treatment regulated UBE2C protein expression (**B**), colony formation (**C**, upper panel), cell density (**C**, lower panel), and cell survival (**D**) in Hela cell. (**E**) Flow chart for the in vivo experimental design and treatment schedule. (**F**–**I**) Quantification of tumor weights and volumes in mice treated with CCI779. (**J**) Tumor sections were observed by IHC for UBE2C. The liver and kidney were fixed, sectioned, and stained with H&E. (**K**) The protein expression levels of UBE2C, mTOR, AKT, and PI3K were detected by western blotting of tumor tissue extracts. The graphics represent the relative intensity of western blot lanes studied by densitometry. (**L**) Tumor sections were observed by immunofluorescence for UBE2C and mTOR. The positive cells showed fluorescence in tumors treated with CCI779. (**M**) The co-localization in (L) is presented as the product of the differences from the mean (PDM) image. White color pixels indicate co-localization coefficient. A 2.5-dimensional reconstruction and fluorescence intensity of the respective insets in (**A**) compared between green, and red fluorescence. * *p* < 0.05, ** *p* < 0.01, and *** *p* < 0.001. Scale bars 50 μm.

We searched for UBE2C in the drug database and looked for potential drugs for the treatment of CESC. As shown in Figure 6A for nine different types of cancer cell lines, the gene (UBE2C) regulated by the mTOR inhibitor had the same gene-drug correlation trend. CCI779 is an mTOR inhibitor; its anti-cancer activity has been demonstrated in colorectal carcinoma and prostate cancer [28,29]. Therefore, we evaluated the anti-cervical cancer activity of CCI779 using the MTT cell proliferation assay in vitro. CCI779 treatment downregulated UBE2C expression in HeLa cells and HeLa cell proliferation, colony formation and in a dose dependent manner (Figure 6B–D). Chemotherapy to cure cervical cancer is used in clinical practices for treatment. We further evaluated the efficacy of CCI779 in inhibiting cervical cancer in vivo. CCI779 was administered on the fifth day after the xenotransplantation of cancer cells into mice; after injections every 2 d, the tumor significantly stopped growing. This result was reflected in the weight and volume of the tumor (Figure 6F-I). The positive signal of UBE2C after CCI779 treatment was significantly reduced, but it did not affect the weight of the mice, nor liver and kidney toxicity (Figure 6J). To confirm that mTOR inhibitors can regulate the level of UBE2C, we extracted proteins from subcutaneous tumor tissues and showed that CCI779 could reduce the level of UBE2C and affect phosphorylated mTOR/AKT/PI3K (Figure 6K). The results of immunofluorescence staining also indicated that UBE2C (green color) in the CCI779 group was significantly reduced, but it did not affect the total mTOR level (Figure 6L). Furthermore, we computed the overall co-localization inside the tumor sections, and observed that Pearson′s coefficient of the CCI779 was significantly reduced from 0.341 to 0.03 compared to the vehicle group (Figure 6M, left panel). We also confirmed the fluorescence distribution using 2.5-dimensional reconstructions and intensity profiles. In the CCI779 group, we further confirmed that the green and red fluorescence did not fully match that of the vehicle group (Figure 6M). These data showed that UBE2C is a potential anti-cancer target for cervical cancer drug development.

## 4. Discussion

TMA revealed that UBE2C was highly expressed in cervical cancer specimens and the expression levels correlated with BMI values and cervical cancer cell types. In addition, we found the level of UBE2C expression was correlated with HPV 58 infection. In vitro analysis showed that proliferation of HeLa cervical cancer cells was enhanced by overexpression of UBE2C, but it was impaired by knockdown of UBE2C and UBE2C inhibitors. The synergistic activity of UBE2C in chemotherapy drug treatment of HeLa cells was revealed. Moreover, the growth of TC-1 cells in mice was suppressed by treatment with the mTOR inhibitor CCI-779. Our results suggest that UBE2C is a critical factor in HPV-related cervical cancer; thus, it could be a useful novel tumor marker for accurately identifying patients with cervical cancer. In addition, UBE2C may serve as a target for treatment of cervical cancer in the future.

In this study, TMA data demonstrated that a high proportion (89.4%) of cervical cancer cases exhibited a high expression level of UBE2C protein (Supplementary Table S1), which was supported by the gene profiling assay of UBE2C gene expression in cervical cancer tissue [30,31]. Approximately 70% of tumor samples expressed UBE2C at H-score values of 1–100, and 20% expressed UBE2C at higher levels with H-score > 100 (Supplementary Table S1). The level of UBE2C expression was not significantly associated with FIGO or tumor size; it might be an independent prognostic factor in cervical cancer. These data were similar to those observed for cases of breast cancer and nasopharyngeal carcinoma patients [32,33]. In our cervical cancer cases, the UBE2C level was not associated with aggressive progression and poor outcome, which had been revealed in the cases of malignant glioma [34]. Whether the overall survival in women with cervical cancer is associated with extreme BMI values (underweight and overweight) remains controversial [35,36]. Our clinical data showed that UBE2C expression is correlated with BMI; however, the physiological and biological implications should be further investigated. Compared to adenocarcinoma cervical cancer, UBE2C protein expression was more frequently associated with squamous cell carcinoma (SCC); this result was consistent with the TCGA data (Figure 1). 

The E6 protein of high-risk type HPV15, 18, and 58 was detected in both tumor and non-tumor sites of a cervical cancer tissue block, but the E6 expression level was significantly higher in the tumor site (Figure 3 and Figure 4). In addition, HPV co-infection was found in our cases, and the occurrence of multiple infections seemed common, which was supported by previous reports [26,27]. Although, the HPV coinfection genotype occurred at random and independent of cervical disease [26], we believe that the clinical implication of the HPV genotype coinfection and cervical disease remains to be further elucidated. A high HPV-58 infection rate was noticed in tumor cases (Figure 3); these data were also supported by previous studies in Taiwan and Korea [9]. Together, these results suggest that the high association between HPV-58 and developing cervical cancer supports the importance of identifying the HPV-58 genotype in the management of women who are HPV-positive. The HPV 58 genotype should be included in HPV vaccination regimens, especially in countries with high prevalence [37,38]. 

We found that the level of UBE2C expression was correlated with the expression of HPV16, 18, and 58 E6 (Supplementary Table S2; Figure 2). Similarly, the microarray analysis showed that UBE2C was one of the molecular signature genes in HPV-induced carcinogenesis [39]. Another study also revealed that the expression of the UBE2C gene was downregulated in HPV18 E6 knockdown HeLa cells [40]. Together, these data indicated that HPV infection regulates UBE2C expression in carcinogenesis. We evaluated the role of UBE2C in HPV associated cervical cancer cell lines in which proliferation was impaired by UBE2C knockdown or enhanced by UBE2C overexpression (Figure 5). The results of these studies were also supported by decreased cell proliferation in UBE2C knockdown cells in cases of nasopharyngeal carcinoma and breast cancer [22,33]. The mTOR inhibitor CCI779 showed anti-cancer activity in colorectal carcinoma and prostate cancer cases [28,29]; here, we also showed the anti-cervical cancer effects of CC1779 in vitro and in vivo (Figure 6). 

Regarding the mechanism by which CCI-779 suppresses UBE2C levels, previous studies clearly indicate that CCI-779 disrupts the UBE2C enhancer and recruits coactivators [29] to the UBE2C promoter. Many previous studies have reported that mTOR blockers inhibit mitochondrial gene transcription through multiple mechanisms that disrupt protein interactions between the transcriptional coactivator Yin Yang 1 1 (YY1), resulting in reduced recruitment of PGC-1α to the mitochondrial gene promoter [41]. In addition, rapamycin can block the expression of sterol regulatory element binding protein 1 (SREBP1) target genes by inhibiting the nuclear accumulation of SREBP1 [42]. In our results, it was also confirmed that CCI-779 reduced the stability of UBE2C protein in a concentration-dependent manner (Figure 6), suggesting that the direct downregulation of UBE2C protein levels induced by CCI-779 is caused by UBE2C, resulting from reduced mRNA transcription and stability. Although mTOR inhibitors have shown great potential as antitumor agents, and CCI-779 has been approved by the US Food and Drug Administration (FDA) as a first-line treatment for patients with advanced refractory renal cell carcinoma [43].

## 5. Conclusions

**Figure 7:**
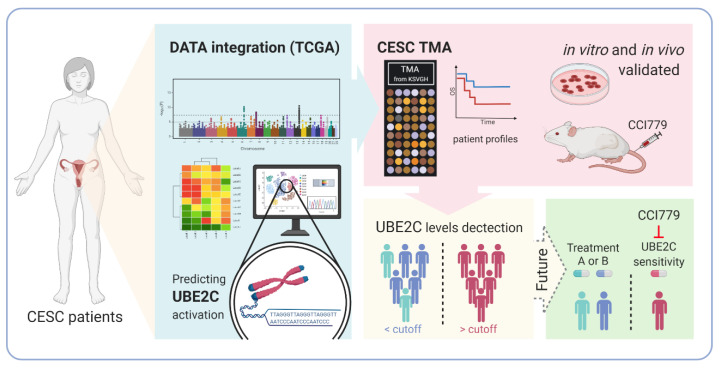
Proposed model of UBE2C regulation and prediction during tumor progression.

In summary, those CSC patients with high UBE2C expression may have better CCI-779 treatment response than those with low UBE2C expression. Further research is needed to investigate whether UBE2C could be used as a biomarker to predict CCI-779 treatment response in CESC patients (Figure 7).

## Data Availability

Medicine, Kaohsiung Veterans General Hospital for the technical assistance.

## Supplementary Materials

The following are available online at https://www.mdpi.com/2218-273X/11/1/37/s1, Table S1: Clinicopathological characteristics andACSS2 expression in patient cervical sample. Table S2: Tumor markers, UBE2C and HPV expression in patient cervical cancer sample.
