# Supplementary material for: UBE2C Drives Human Cervical Cancer Progression and Is Positively Modulated by mTOR
Source: Biomolecules. 2020 Dec 30;11(1):37. doi: 10.3390/biom11010037 (PMC7823929; doi:10.3390/biom11010037)
Supplement: Supplementary file 1 [file biomolecules-11-00037-s001.pdf]

Supplementary table 1. Clinicopathological characteristics andACSS2 expression in patient cervical sample.

Characteristics	ACSS2<br>H-score >100 / n / % /Median	UBE2C<br>H-score = 201-300 / n(%)/Median	χ^2/p-value
**FIGO**			
IA1	0 (0) / -	0 (0) / -	0.976/-
IA2	1 (0.3) /120	0 (0) / -	
IB1	15 (5.1) / 135	5 (1.7) /255	
IB2	6 (2.0) / 142.5	3 (1.0) / 240	
IIA1	2 (0.7) / 135	0 (0) / -	
IIA2	1 (0.3) / 120	2 (0.7) / 232.5	
IIB	4 (1.4) / 140	4 (1.4) / 247.5	
IIIA	0 (0) / -	0 (0) / -	
IIIB	4 (1.4) /165	2 (0.7) /225	
IVA	1 (0.3) / 150	1 (0.3) / 210	
IVB	1 (0.3) / 120	0 (0) /-	
Unknown	3 (1.0) / 120	0 (0) /-	
**TNM Stage_Primary Tumor (T)**			
T1a	2 (0.7) / 120	0 (0) / -	0.393/-
T1b	16 (5.4) / 142.5	7 (2.4) / 240	
T2a	7 (2.4) / 120	3 (1.0) / 240	
T2b	8 (2.7) / 135	3 (1.0) / 255	
T3	4 (1.4) / 165	1 (0.3) / 240	
T4	1 (0.3) / 150	3 (1.0) / 210	
**TNM Stage_Regional Lymph Nodes (N)**			
N0	27 (9.2) / 135	7 (2.4) / 240	0.06/-
N1	11 (3.7) / 135	10 (3.4) / 240	
NX	0 (0) / -	0 (0) / -	
**TNM Stage_distant Metastasis (M)**			
M0	29 (10) / 120	12 (4.2) / 240	0.643/-
M1	4 (1.4) / 135	2 (0.7) / 255	
Mx	5 (1.7) / 150	3 (1.0) / 225	

FIGO, theinternational Federation of Gynecology and Obstetrics; TMN, tumor-node-metastasis; p<0.05 *,p<0.005**,p<0.001***

Supplementary table 2. Tumor markers, UBE2C and HPV expression in patient cervical cancer sample.

Characteristics	NO. (%)	Median/Mean	UBE2C H-score = 0/n (%)	UBE2C H-score = 1-100 / n (%)	UBE2C H-score = 101-200 n (%)	UBE2C H-score = 201-300 n (%)/	χ^2/p-value
**CA125**							
<35	75 (25.5)	14.80/15.9.0	7 (2.4) / 0	53 (18.0) / 15	10 (3.4) / 127.5	5 (1.7) / 240	0.99
>35	42 (14.3)	82.15/299.34	5 (1.7) / 0	30 (10.2) / 22.5	5 (1.7) / 150	2 (0.7) / 225	
Unknown	177 (60.2)	-/-	19 (6.5) / 0	125 (42.5) / 20	23 (7.8) / 135	10 (3.4) / 240	
**CA19-9**							
<37	57 (16)	10.60/13.95	7 (2.4) / 0	47 (16.0) / 18	7 (2.4) / 135	4 (1.4) / 240	0.96
>37	33 (14)	92.43/326.97	5 (1.7) / 0	25 (8.5) / 15	4 (1.4) / 127.5	0 (0) / -	
Unknown	204 (66)	-	19 (6.5) / 0	136 (46.3) / 20	27 (9.2) / 135	13 (4.4) / 240	
**CEA**							
<5	170 (57.8)	1.85/2.21	23 (7.8) /0	153 (52.0) / 20	28 (9.5) / 127.5	14 (4.8) / 240	0.98
>5	54 (18.4)	9.18/44.35	6 (2.0) / 0	37 (12.6) / 20	8 (2.7) / 127.5	9 (1.0) / 240	
Unknown	70 (23.8)	-	2 (0.7) / 0	18 (6.1) / 20	2 (0.7) / 165	0 (0) / -	
**SCC**							
<1.5	119 (40.5)	0.7/0.71	11 (3.7) / 0	86 (29.3) / 15	16 (5.4) / 142.5	6 (2.0) / 240	0.93
1.5-5	54 (18.4)	2.6/2.8	6 (2.0) / 1	37 (12.6) / 20	9 (3.1) / 120	2 (0.7) / 232.5	
>5	67 (22.8)	16/21.16	9 (3.1) / 0	54 (18.4) / 22.5	9 (3.1) /150	7 (2.4) / 240	
Unknown	54 (18.4)	-	5 (1.7) / 0	31 (10.5) / 18	4 (1.4) / 120	2 (0.7) / 247.5	
**HPV16 H-score**							
0	0 (0)	-/-	0 (0) / -	0 (0) / -	0 (0) / -	0 (0) / -	0.15
1-100	90 (30.6)	80/74.77	13 (4.4) / 0	62 (21.1) / 20	8 (2.7) / 127.5	7 (2.4) / 240	
101-200	60 (20.4)	160/156.32	6 (2.0) / 0	47 (16.0) / 17.5	4 (1.4) / 150	3 (1.0) / 240	
201-300	144 (49.0)	270/263.71	12 (4.1) / 0	99 (33.7) / 20	26 (8.8) / 127.5	7 (2.4) / 240	
**HPV18 H-score**							
0	3 (1.0)	0/0	0 (0) / -	1 (0.3) / 60	0 (0) / -	2 (0.7) / 225	0.14
1-100	77 (26.2)	80/77.29	14 (4.8) / 0	51 (17.3) / 10.5	7 (2.4) / 150	5 (1.7) / 255	
101-200	154 (52.4)	160/155.25	13 (4.4) / 0	111 (37.8) / 20	21 (7.1) / 135	9 (3.1) / 240	
201-300	60 (20.4)	240/244.68	4 (1.4) / 1	45 (15.3) / 25	10 (3.4) / 127.5	17 (5.8) / 210	
**HPV58 H-score**							
0	0 (0)	-/-	0 (0) / -	0 (0) / -	0 (0) / -	0 (0) / -	5.4367E-08/ ***
1-100	47 (16.0)	80/73.51	12 (4.1) / 0	33 (11.2) / 10	1 (0.3) / 120	1 (0.3) / 225	
101-200	167 (56.8)	160/154.20	14 (4.8) / 0	131 (44.6) / 20	18 (6.1) / 120	4 (1.4) / 232.5	
201-300	80 (27.2)	240/236.18	5 (1.7) / 0	44 (15.0) / 33.75	19 (6.5) / 150	12 (4.1) / 240	

CA125, cancer antigen 125; CA19-9, cancer antigen 19-9; CEA, carcinoembryonic antigen; SCC, Squamous Cell Carcinoma antigen; UBE2C, Ubiquitin-conjugating enzyme E2C; HPV, Human Papillovirus
